# A steam-based method to investigate biofilm

**DOI:** 10.1038/s41598-018-31437-y

**Published:** 2018-08-29

**Authors:** Jason Tasse, Andréa Cara, Maude Saglio, Régis Villet, Frédéric Laurent

**Affiliations:** 1 CIRI – Centre International de Recherche en Infectiologie, Inserm, U1111, Université Claude Bernard Lyon 1, CNRS, UMR5308, Ecole Normale Supérieure de Lyon, Univ Lyon, Lyon, France; 2 Institut des Sciences Pharmaceutiques et Biologiques de Lyon, Université Claude Bernard Lyon 1, Lyon, France; 3 Institut des Agents Infectieux, Hospices Civils de Lyon, Lyon, France; 4 Present Address: BIOASTER, 40 avenue Tony Garnier, 69007 Lyon, France

## Abstract

Biofilm has become a major topic of interest in medical, food, industrial, and environmental bacteriology. To be relevant, investigation of biofilm behavior requires effective and reliable techniques. We present herein a simple and robust method, adapted from the microplate technique, in which steam is used as a soft washing method to preserve biofilm integrity and to improve reproducibility of biofilm quantification. The kinetics of steam washing indicated that the method is adapted to remove both planktonic bacteria and excess crystal violet (CV) staining for *S. aureus*, *S. epidermidis*, *S. carnosus*, *P. aeruginosa*, and *E. coli* biofilm. Confocal laser scanning microscopy confirmed that steam washing preserved the integrity of the biofilm better than pipette-based washing. We also investigated the measurement of the turbidity of biofilm resuspended in phosphate-buffered saline (PBS) as an alternative to staining with CV. This approach allows the discrimination of biofilm producer strains from non-biofilm producer strains in a way similar to CV staining, and subsequently permits quantification of viable bacteria present in biofilm by culture enumeration from the same well. Biofilm quantification using steam washing and PBS turbidity reduced the technical time needed, and data were highly reproducible.

## Introduction

Biofilm is defined as a community of microorganisms attached to a solid surface and embedded in a matrix facilitating the survival of bacteria in hostile environments. They are involved in a wide variety of biogeochemical cycling processes in water, soil, sediment and subsurface environments, but also have biotechnological applications such as filtration of drinking water, degradation of wastewater and solid waste, and may be used as biocatalysis in the production of bulk and fine chemicals as well as biofuels. They are also responsible for persistent contamination in the food industry, impacting the safety and quality of products^1^. In addition, they are now recognized as a major virulence factor in infections of plants and animals, including humans, especially in the presence of medical devices or implants^2,3^.

Since its first description in 1978 by Costerton *et al*.^4^, multiple methods have been proposed to investigate biofilms using *in vitro* models, ranging in complexity from bacterial colony growing on specific media^5^ to complex continuous culture bioreactor systems^6^. One of the most commonly used biofilm models for high-throughput analyses is based on 96-well microtiter plates^7,8^. This historical device is adaptable to most bacterial or fungal species, and remains widely used to compare the behavior of bacterial biofilms, screen for potential anti-biofilm agents, and identify genes involved in biofilm formation in liquid media^9^. In the microplate-based method, cells are grown for a specific period before being washed in order to remove planktonic bacteria. The biofilm formed can then be assessed using various approaches including staining with a dye (Crystal Violet – CV, Safranin Red, or Congo Red) for total biomass quantification^10^, growth ability (colony forming units) for viable cell enumeration^11^, and specific fluorescent probes (FITC, SYPRO Ruby, Calcofluor White) for the quantification of extracellular polymeric components^12^. For the staining protocol using CV, both living and dead bacteria as well as the matrix are colored. Dyes are then solubilized using solvents such as 33% glacial acetic acid, dimethyl sulfoxide (DMSO), or 95% ethanol, depending on the organism, and the optical density (OD) measured to quantify biofilm formation^13^. An alternative method has been proposed in which biofilm is resuspended in phosphate-buffered saline (PBS) instead of staining^14,15^, yet, to the best of our knowledge, no comparison with the classical staining method has been performed.

All these methods have several limitations. The main one is the lack of reproducibility of the results notably due to aggressive pipette-based washing^16^, which is likely to cause the random detachment of large amounts of biofilm. Furthermore, the native structure/spatial organization of the biofilm may also be modified leading to skewed measurements, and once the biofilm is stained it is no longer possible to determine the number of viable cells of that well. More generally, dyes are toxic, mutagenic, and/or irritant for the experimenter, environmentally hazardous, and require protective equipment^17^. In this context, we propose a new method in which steam is used as a soft washing method to preserve biofilm integrity and to improve reproducibility of biofilm quantification. We also investigated the measurement of turbidity to quantify biofilm matrix and bacteria as an alternative to staining.

## Results

### Steam washing concept

**Figure 1:**
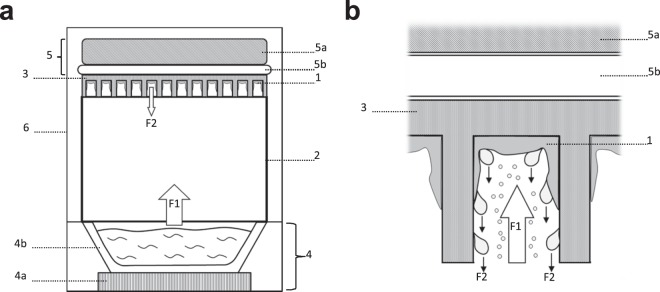
Steam washing process design. (**a**) Cross sectional view of steam washing system. After 24-h formation of the biofilm in the microplate, the liquid media is removed by inversion and the microplate is hung upside down in the system. The biofilm wash system (1) is composed of a holding device (2) configured to hold a microplate (3). A steam generating system (4), composed of a heating unit (4a) and a reservoir (4b) creating a flow of steam (F1) leading to the formation of droplets (F2) by condensation that removes non-adherent bacteria and media. To enhance condensation and protect bacteria of the biofilm from heat, a cooling system (5), composed of a cooling device (5a) and a thermal transmission device (5b) is in contact with the microplate. A semi-closed cover (6) enhances condensation. (**b**) Enlarged view of a microplate well in the wash system.

**Figure 2:**
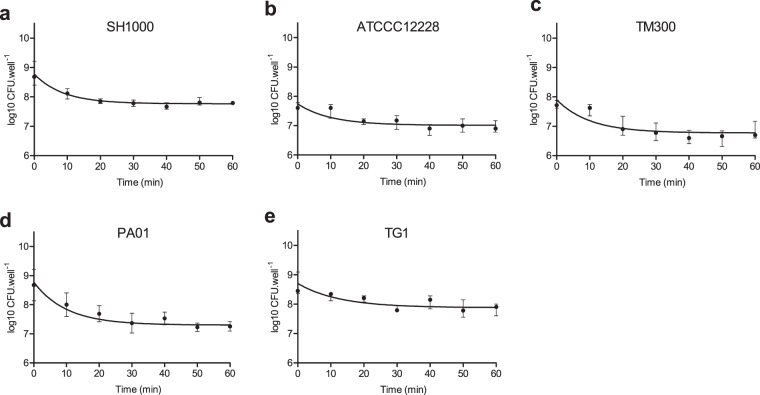
Steam washing kinetics for removal of the planktonic bacteria. A 24h-old biofilm was formed by (**a**) SH1000 (*S. aureus*), (**b**) ATCC 12228 (*S. epidermidis*), (**c**) TM300 (*S. carnosus*), (**d**) PA01 (*P. aeruginosa*), and (**e**) TG1 (*E. coli*) which were washed using the steam method. Viable cell count was measured every 10 min over 1 h. Data represent the median and interquartile range of three experiments performed in triplicate.

The new washing method is based on water vapor condensation. To remove planktonic bacteria without altering biofilm integrity, the support (microplate) on which biofilm has formed needs to be placed upside down above a source of steam and the bottom to be in contact with a cooling system to favor condensation and preserve viable bacteria (Fig. 1a,b). Water droplets form and accumulate in each well on the biofilm surface before falling back down along with the non-adherent bacteria. This system can be easily adapted in any laboratory by using boiling water to produce the steam, and a cooling system (a simple ice pack for transport being adequate) to chill the microplate. This soft washing process was initially tested on a 24 h-old biofilm of an *S. aureus* strain (SH1000) formed in a 96-well plate. Viable cell count was measured every 10 min over 1 h to determine the time needed to correctly remove planktonic bacteria (Fig. 2a). Results indicate that a plateau was reached after 35 min, corresponding to the minimal time required to ensure maximal biofilm wash for this strain. During the washing process, the spreading of bacteria was investigated by taking multiple samples from the various parts of the steam washing system (steam generating system, reservoir, water, holding device) and around it; no dissemination of viable bacteria was found which confirmed the safety of the system. Of note, water drops containing the washed planktonic bacteria fall back into the boiling water reservoir, that allow the destruction of viable bacteria.

The homogeneity of the steam washing between wells of the same microplate was investigated. For this, the mean numbers of viable bacteria among wells of the same row (and then the same column) were compared using one-way analysis of variance (ANOVA); the null hypothesis was that the biofilm formed was identical between rows (and between columns). Results indicated that the biofilm bacteria count was similar in both rows (*p* = 0.1452, Supplementary Fig. 1a) and columns (*p* = 0.1183, Supplementary Fig. 1b).

### CV assay using steam washing

**Figure 3:**
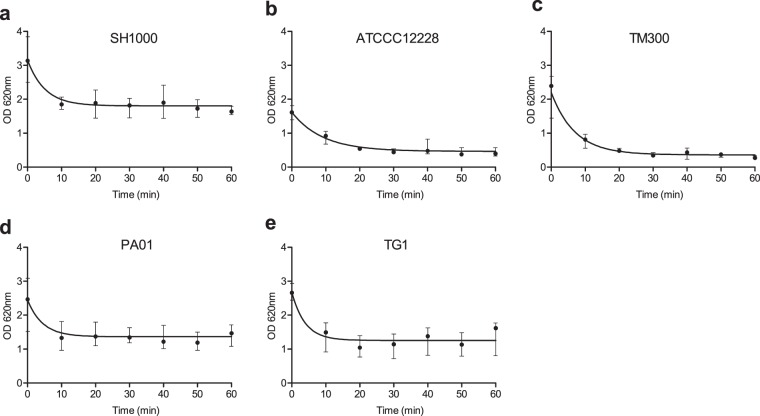
Steam washing kinetics for removal of excess stain. A 24h-old biofilm was formed by (**a**) SH1000 (*S. aureus*), (**b**) ATCC 12228 (*S. epidermidis*), (**c**) TM300 (*S. carnosus*), (**d**) PA01 (*P. aeruginosa*) and (**e**) TG1 (*E. coli*) which were washed using the steam method before staining by crystal violet. The capacity of the steam washing to remove excess stain was evaluated by measuring OD_620_ every 10 min over 1 h. Data represent the median and interquartile range of three experiments performed in quadruplicate.

The steam washing method therefore appears adapted to the removal of planktonic bacteria. In this context, we tested the possibility to combine the steam washing method with the CV assay. For this, three reference strains described as a biofilm producer (*S. aureus* SH1000) or a non-biofilm producer (*S. epidermidis* ATCC 12228 and *S. carnosus* TM300) were used. The time needed to remove planktonic bacteria on ATCC12228 and TM300 using steam was determined as previously done for SH1000; it was found that a plateau was reached after 40 min for both strains (Fig. 2b,c). The biofilms were then stained using CV, and excess stain was removed by steam washing. The time needed to completely remove excess CV was assessed by measuring OD_620_ every 10 min over 1 h; it was found that a plateau was reached after 30 min (Fig. 3a–c).

**Figure 4:**
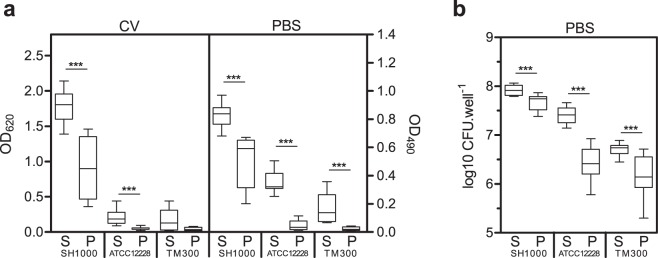
Application of steam washing process. (**a**) Biofilm quantification by crystal violet (CV) coloration (OD_620_) or PBS turbidity (OD_490_) for SH1000 (*S. aureus*), ATCC 12228 (*S. epidermidis*), TM300 (*S. carnosus*), PA01 (*P. aeruginosa*), and TG1 (*E. coli*). Biofilms were washed using the steam (S) or pipette-based method (P). Data are presented as box plots of three experiments performed in quadruplicate. (**b**) Viable cell count of bacteria assessed after PBS turbidity. Data are presented as box plots of three experiments performed in quadruplicate. ***P value < 0.001, as determined by the Mann-Whitney U test.

The CV assay using the steam washing method was then compared to the classical CV assay using pipette-based washing. Staining for all strains was greater using steam washing (Fig. 4a) which is likely to be related to the soft washing process (see below for Confocal Laser Scanning Microscopy (CLSM) confirmation). Interestingly, we noticed that the standard deviation for the SH1000 strain was lower using steam washing (0.234 vs. 0.441) which indicates that the reproducibility of this technique was better than pipette-based washing. Interestingly, as control wells without bacteria were prepared and ODs were subtracted from the values for strain samples, the data suggest that ATCC 12228 and TM300 were not strictly non-biofilm producers, and have a residual (even low) ability to form weak biofilm.

To explore the capacity of steam washing to be used with other species, *Pseudomonas aeruginosa* PA01 and *Escherichia coli* TG1, two isolates known to be biofilm producer strains, were tested under the same conditions as SH1000. The kinetics for the removal of planktonic bacteria and excess stain with steam found that a plateau was reached after 45 min (Fig. 2d,e) and 20 min (Fig. 3d,e), respectively, for both strains. As for *S. aureus*, comparison with pipette-based washing was performed and found a significantly greater staining with lower standard deviation using steam washing for PA01 (Supplementary Fig. 2a). Interestingly, for TG1 there was no significant difference between the washing methods (Supplementary Fig. 2a) suggesting that TG1 biofilm was more resistant to the aggressiveness of pipette-based washing.

To conclude, all washing kinetics indicate that bacteria were effectively removed until a plateau was reached. This was reproducible and was observed for all bacteria tested, strongly suggesting that the final balance achieved corresponded to the removing of non-adherent bacteria, and that the greater amount of biofilm measured using the new method is not a consequence of a partial wash.

### Biofilm quantification using PBS turbidity

We then hypothesized that biofilm formation could be measured without a staining step by measuring the turbidity of a biofilm resuspended in PBS. First, an absorbance spectrum was determined using steam-washed biofilms of the strains investigated (SH1000, ATCC 12228, TM300, PA01, and TG1); this found that OD measured at a wavelength 490 nm allowed the discrimination of the strains with a high measure amplitude, and that control wells containing PBS had a low OD (Supplementary Fig. 3). Using PBS turbidity (OD_490_), we were able to discriminate staphylococcal biofilm producer strains from non-producer strains in a way similar to that when CV staining and OD_620_ were used (Fig. 4a, Supplementary Fig. 4). It is of note that biofilm production of the ATCC12228 strain was less important regarding the OD_620_ than regarding OD_490_ when steam washing was used. This could be explained by the additional step needed to add CV to the well using a pipette, that can lead to detachment of the biofilm (as is the case during pipette-based washing).

Regarding *P. aeruginosa* PA01 and *E. coli* TG1, we observed that the OD obtained using PBS turbidity was lower than CV staining for both washing methods (Supplementary Fig. 2). This could be explained in part by the nature/composition of the extracellular matrix which is different from staphylococcal species; *P. aeruginosa* is characterized by a high content of alginate, and *E. coli* by a high content of cellulose. This composition probably induced a high capture of CV, leading to high OD_620_ values, but does not induce a high turbidity when the biofilm was resuspended in PBS, leading to low OD_490_ values. However, even if the OD_490_ values were low, both OD_620_ and OD_490_ allowed the classification of PA01 and TG1 as strong biofilm producers according to the method described by *Stepanović et al*.^10^ (data not shown).

Interestingly, using the non-bactericidal protocol of PBS turbidity for biofilm quantification, viable cell count can be performed on the same well by subsequently plating the bacterial suspension. Results showed that, when steam washing was used, the bacterial counts were significantly higher than those obtained using pipette-based washing for all strains, except *P. aeruginosa* PA01 and *E. coli* TG1 for which the count was not significantly different (Fig. 4b, Supplementary Fig. 2b). Again, the standard deviations for all strains were lower using steam washing, indicating that the reproducibility was better than for pipette-based washing, except for *E. coli* TG1 for which the reproducibility was similar.

In addition, the better reproducibility and the ability to discriminate high from low biofilm producers using PBS turbidity for staphylococcal species was confirmed by using a set of 5 clinical *S. aureus* strains (Supplementary Fig. 5a,b). Finally, the impact of growth media on steam wash was investigated using BHIg and BHI media and showed that they influence the time to reach the plateau (Supplementary Fig. 6). These results indicate that any modification of the parameters used to form the biofilm may impact the time for washing.

### Biofilm structure after steam washing

**Figure 5:**
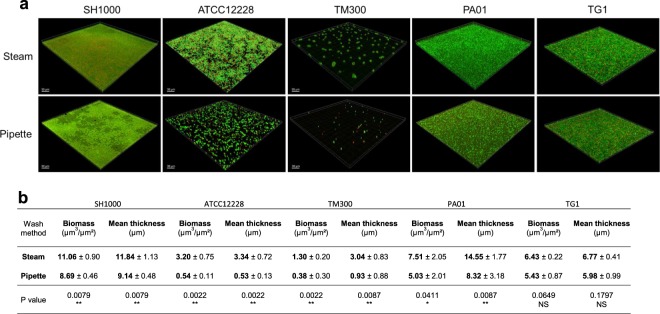
Confocal laser scanning microscopy (CLSM) of bacteria biofilm washed using the steam or pipette-based method. (**a**) Images are 3D visualizations of representative biofilm structures washed using the steam (top) or pipette-based method (bottom) stained with Syto-9 and PI for SH1000 (*S. aureus*), ATCC 12228 (*S. epidermidis*), TM300 (*S. carnosus*), PA01 (*P. aeruginosa*), and TG1 (*E. coli*). Images represent an area of approximately 320 × 320 µm. (b) Quantification of biomass and mean thickness of the biofilm measured by CLSM using Comstat2. *P value < 0.05, **P value < 0.01, as determined by the Mann-Whitney U test.

To confirm that bacteria in biofilm were not killed by the steam washing step, biofilms were examined using CLSM and Live/Dead staining. Qualitative image analysis found that bacteria were alive (green) irrespective of the washing method (Fig. 5a). Furthermore, the morphology of the biofilm was preserved with steam washing, while holes and alterations were present within the biofilm after pipette-based washing (strain SH1000, Fig. 5a). The biomass and mean thickness calculated using Comstat2 software were significantly greater for steam washing than for pipette-based washing for all strains except TG1, for which they were not significantly different (Fig. 5b). These data confirmed that steam washing preserved the integrity of the biofilm better than pipette-based washing that leads to detachment of thin and fragile biofilm such as that produced by ATCC12228 and TM300. For *E. coli* TG1, the absence of significant difference between the washing methods strongly suggests that *E. coli* TG1 biofilm was robust enough to resist the aggressiveness of pipette-based washing. Taken together, these data confirmed that steam washing was adapted to the study of both robust and fragile biofilm from different species.

### Reproducibility of steam washing and PBS turbidity combined

**Figure 6:**
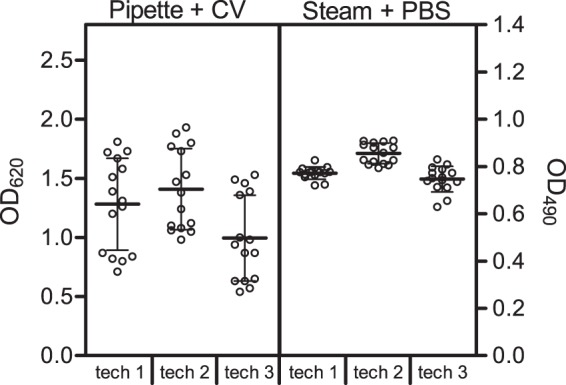
Reproducibility of crystal violet (CV) staining using the pipette-based method and PBS turbidity using the steam method among 3 technicians. Biofilm quantification by CV staining (OD_620_) or PBS turbidity (OD_490_) for SH1000 (*S. aureus*). Data represent three experiments in performed in quadruplicate.

Finally, the reproducibility of CV staining using pipette-based washing and PBS turbidity using steam washing as performed by three distinct technicians was investigated using *S. aureus* SH1000. The standard deviations were lower using steam washing and turbidity measurement than when using pipette-based washing and CV staining for all three technicians (0.026, 0.043, and 0.054 vs. 0.389, 0.343, and 0.363; Fig. 6) indicating that the increased reproducibility of PBS turbidity using steam wash is conserved between technicians. Furthermore, the technical time required to perform each of the methods on 4 or 96 wells was measured for each technician (the time during which the microplate was on the steam system was not included as this step is performed without any technician intervention). The PBS turbidity/steam washing method was time saving compared to CV staining using pipette-based washing (−17.5% and −40.2% respectively, Supplementary Table 1).

### Perspectives

The antimicrobial susceptibility of biofilms using steam washing was not evaluated in the present study. This approach could theoretically be performed using this new method, but the time required to completely remove the antimicrobial agent by steam needs to be determined. Regarding the Calgary Biofilm Device^18^, steam washing is probably not adapted to remove non-adherent bacteria from pegs because the cooling system would not be able to chill the extremity of the peg as much as the bottom of a microplate. Furthermore, there is probably a risk of cross-contamination from peg to peg in this setup because bacteria and biofilm are on the outer side of the pegs and not inside a well.

## Conclusion

We propose herein two ways to easily improve the study of biofilms; first a new method for microtiter plate assays to softly wash the biofilm with steam instead of pipetting, and second the quantification of the biofilm using PBS turbidity instead of staining with a dye. These processes can be easily adapted in all laboratories and at low cost. They require less technical time, and reduce steps involved which leads to highly reproducible data compared to the methods used up to now. As the steam washing is less aggressive than pipette-based washing, this new method is going to make it possible to investigate thin, early, and/or fragile biofilms, as described here for strains ATCC 12228 and TM300. To conclude, steam washing is a simple, rapid, inexpensive and robust method which has all the features needed to replace the techniques currently used to study biofilms in medical, environmental, and industrial fields.

Patent of the method: PCT/FR2017/052646.

## Methods

### Bacterial strains

*S. aureus* strain SH1000 (biofilm producer)^19^, *S. epidermidis* strain ATCC 12228 (non-biofilm producer)^20^, *S. carnosus* strain TM300 (non-biofilm producer)^16^, *E. coli* TG1 (biofilm producer), *P. aeruginosa* PA01 (biofilm producer), and 5 clinical *S. aureus* strains responsible for prosthetic joint infections were used in this study.

### Biofilm experiments

All strains were first subcultured on Colombia blood agar plates (bioMérieux) overnight at 36 °C. Brain Heart Infusion (BHI, Becton Dickinson) was inoculated with 3 colonies and incubated 24 h at 36 °C. Inoculum was then standardized to OD_600_ = 1 ± 0.05 and diluted 1:100 with sterile BHI (for *E. coli*) or BHI plus 1% glucose (Biosolve) to obtain a final suspension of 1.5 × 10^7^ UFC.mL^−1^. Aliquots of 200 μL per well were then added to a 96-well polystyrene microplate (Greiner) and incubated 24 h at 36 °C in a humid chamber. Negative control wells were prepared with medium alone. After incubation, plates were first inverted and a quick top-down movement was used to remove growth medium and most of the planktonic bacteria. For pipette-based washing, 200 µL of PBS 1x (Thermo Fisher Scientific, Gibco) were carefully added to the wells using a pipette, and then removed by inversion of the plate. This step was repeated twice. For steam washing, inverted microplates were placed above a steam generating system in contact with a cooling device. The process was semi-covered to enhance condensation (Fig. 1a,b). During the process, the heating unit was set to an appropriate level so as that water vapor was produced. The ice pack used as a cooling device was still cold at the end of the experiment. Microplates were then recovered and tapped on absorbent paper to remove excess liquid accumulated by condensation. To evaluate the formation of biofilms, two approaches were tested. For CV staining, 150 µL of dye (ELITechGroup) were added per well and incubated at room temperature for 5 min. Excess stain was washed using the pipette-based or steam method as described above. Plates were air-dried and the remaining dye was then resolubilized with 200 µL of 33% (v/v) glacial acetic acid per well. Staining (OD_620_) was measured using a micro ELISA Auto Reader, Model 680 (BioRad). For PBS turbidity, the biofilm was resuspended in 200 µL of PBS 1X and homogenized with a tip. Then, the microplate was sealed with a film (Dutscher) and put in an ultrasound bath (Bactosonic) for 10 min (40 Hz) in order to detach trapped bacteria and to remove clusters, following which the turbidity (OD_490_) was measured. The control OD (wells with medium alone) was subtracted from the values for strain samples for all conditions. Bacterial count was assessed on the same wells that underwent biofilm PBS turbidity evaluation, by plating serial dilutions onto Tryptic Soy agar (TSA) plates (bioMérieux) and incubation for 24 h at 36 °C. For an individual experiment four wells were used for each strain, and the experiment was repeated three times. Differences between steam washing and pipette-based washing were assessed using the Mann-Whitney U test with Prism 5.03 software (Prism, GraphPad Software, San Diego California USA). The significance threshold used was P < 0.05.

### Kinetics of steam washing to optimize the removal of planktonic bacteria

To determine the time required to remove planktonic bacteria, the kinetics of steam washing was investigated for all reference strains. A 24-h old biofilm was prepared in seven microplates as described above and then washed with steam for 10, 20, 30, 40, 50, and 60 min. The biofilms were resuspended in 200 µL of PBS 1x, the microplate was sealed and sonicated for 10 min (40 Hz), following which the turbidity (OD_490_) was measured. The bacterial count of 3 wells for each condition was measured by plating serial dilutions onto TSA plates and incubated 24 h at 36 °C. The experiment was performed three times.

### Kinetics of steam washing to optimize the removal of excess stain

To determine the time required to remove excess stain, the kinetics of steam washing was investigated for all reference strains on 24-h old biofilm prepared in seven microplates as described above. Planktonic bacteria were removed using steam and the remaining biofilm was stained with CV. Excess stain was washed with steam for 10, 20, 30, 40, 50, and 60 min, then resolubilized with acetic acid, before the OD_620_ was measured. The experiments were performed three times.

### Equivalent biofilm formation assay

To assess whether, under the experimental conditions used, there was asymmetry in biofilm washing according to the position of the wells on the plate, a test for equivalent biofilm was done on SH1000 strain. A 24-h old biofilm (formed in each of the 96 wells of the microplate) was washed with steam. After resuspension and sonication of the biofilm in PBS 1X, colony counting of each well was performed by plating serial dilutions, grouped by row or column, and compared using one-way ANOVA. If *p* ≤ 0.05, the null hypothesis that biofilm cell counts were equivalent in each row or column was rejected. Conversely, if *p* > 0.05, the biofilm cell density was similar between rows or columns. The experiments were performed three times.

### Confocal laser scanning microscopy

To evaluate the structure of biofilms and to confirm that bacteria were not killed by steam washing, confocal laser scanning microscopy (CLSM) was performed on a 24h-old biofilm of all reference strains. Briefly, a biofilm was formed on 8-well uncoated microslides (iBidi) as described above and washed with either the steam or pipette-based method. The biofilms were stained with Syto9 and PI from the Live/Dead^®^ BacLight^TM^ Bacterial Viability Kit (Molecular Probes) following the manufacturer’s instructions. The biofilms were incubated for 30 min at room temperature in the dark before being rinsed with the steam or pipette-based method. Images were acquired using a Zeiss LSM800, Full GaAsP detectors (Leica Microsystems) CLSM. The biofilms were observed using a 20 × dry lens (Plan-Apochromat 20x/0.8 M27, Leica Microsystems). For both conditions, images were acquired at 2524 × 2524 resolution in at least 5 different regions of each surface analyzed. 3D reconstructions were performed using Imaris (version 8.0) software (Bitplane), and biofilm quantification was performed using Comstat2 (University of Denmark)^21^.

## Electronic supplementary material

Dataset 1
