# Supplementary material for: A steam-based method to investigate biofilm
Source: Sci Rep. 2018 Aug 29;8:13040. doi: 10.1038/s41598-018-31437-y (PMC6115380; doi:10.1038/s41598-018-31437-y)
Supplement: Supplementary file 1 — Dataset 1 [file 41598_2018_31437_MOESM1_ESM.docx]

**A steam-based method to investigate biofilm**

Jason Tasse^1,2,3^, Andrea Cara^1,2,3^, Maude Saglio^1,2,3^, Régis Villet^1,2,3#^, Frédéric Laurent^1,2,3#*^

**Supplementary Information**

**Supplementary Figure 1. Homogeneity of steam wash.** Variability from well to well of a 24 h-old biofilm formed by *S. aureus* strain (SH1000) washed using steam within a 96-wells microplate (based on colony counting). Each point represents the mean of 3 experiments. (a) Results were grouped by row. (b) Results were grouped by column. Statistically significant differences determined by one-way ANOVA mean biofilm should be similar between row (p = 0.1452), and between columns (p = 0.1183).

**a**

**b**

**Supplementary Figure 2. Application of steam washing process.** (a) Biofilm quantification by crystal violet (CV) coloration (OD_620_) or PBS turbidity (OD_490_) for PA01 (*P. aeruginosa*) and TG1 (*E. coli*). Biofilms were washed using steam (S) or pipette (P). Data shown as box plot of three experiments in quadruplicate. (b) Viable cell count of bacteria assessed after PBS turbidity. Data shown as box plot of three experiments in quadruplicate. * P value < 0.05, as determined by the Mann-Whitney U test.

**Supplementary Figure 3.** Absorbance spectrum of a 24 h-old steam-washed biofilm formed by SH1000 (black circle), ATCC 12228 (empty circle), PA01 (black down-pointing triangle), TG1 (empty down-pointing triangle) and TM300 (black up-pointing triangle). PBS alone was represented by empty up-pointing triangle.

**
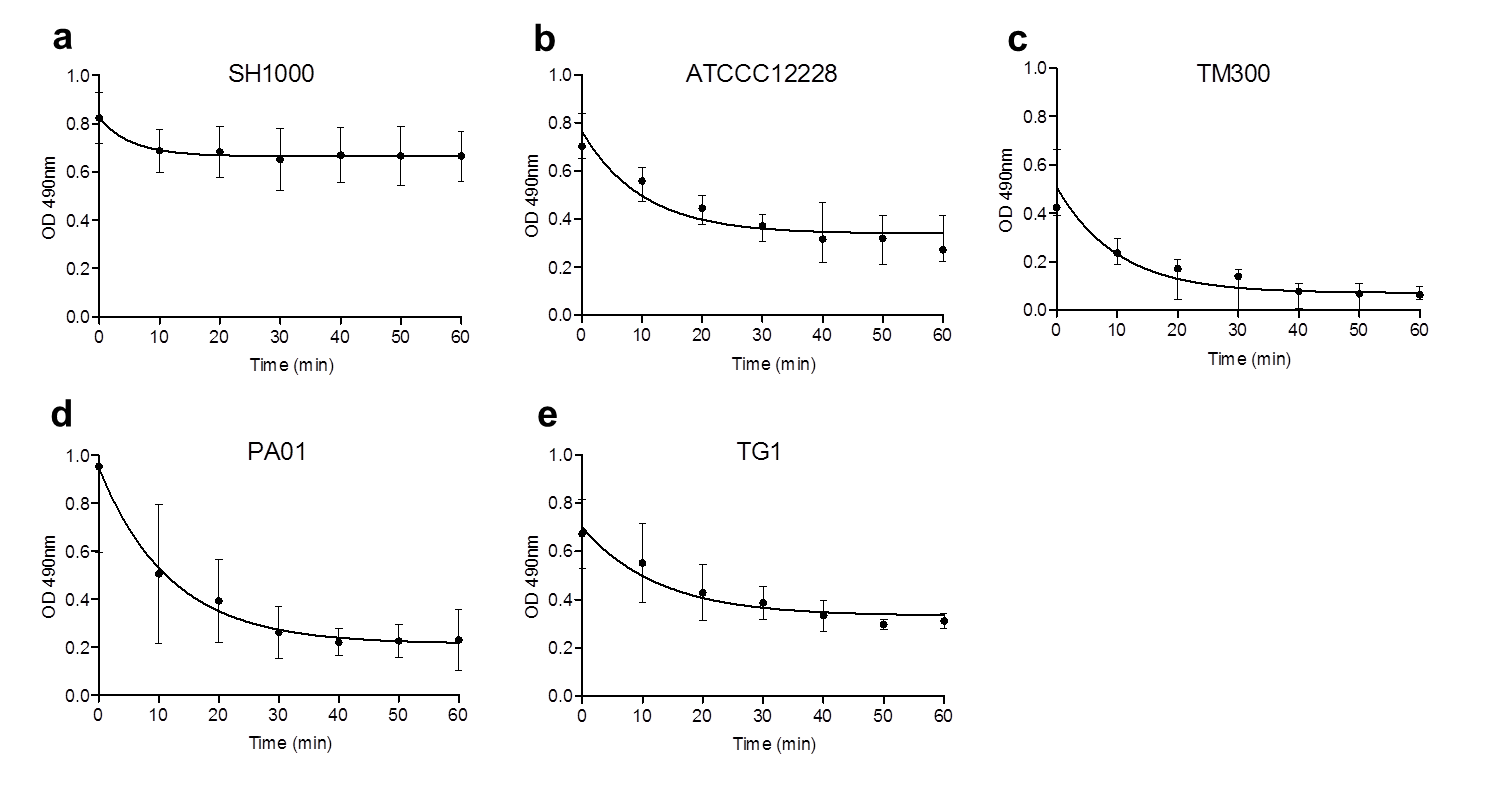
**

**Supplementary Figure 4. Steam washing kinetic to remove planktonic bacteria.** A 24h-old biofilm was formed by (a) SH1000 (*S. aureus)*, (b) ATCC 12228 (*S. epidermidis*), (c) TM300 (*S. carnosus*), (d) PA01 (*P. aeruginosa*) and (e) TG1 (*E. coli*) and wash using steam method. OD_490_ was measured every 10 min over 1 h. Data represent the median and interquartile range of three experiments in triplicate.


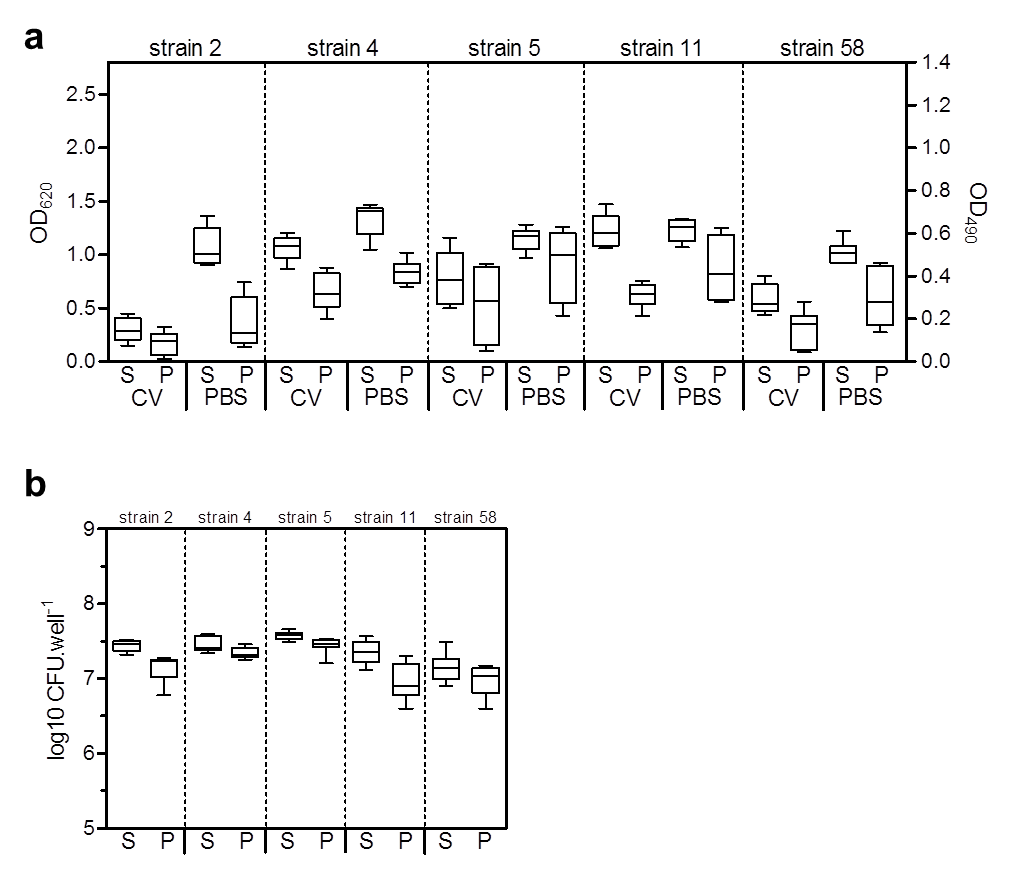


**Supplementary Figure 5. Application of steam washing process on clinical *S. aureus* isolates**. (a) Biofilm quantification by crystal violet (CV) coloration (OD_620_) or PBS turbidity (OD_490_) on five clinical *S. aureus* strains isolated from patients with prosthetic joint infections. Biofilms were washed using steam (S) or pipette (P). Data shown as box plot of three experiments in triplicate. (b) Viable cell count of bacteria assessed after PBS turbidity. Data shown as box plot of three experiments in triplicate.


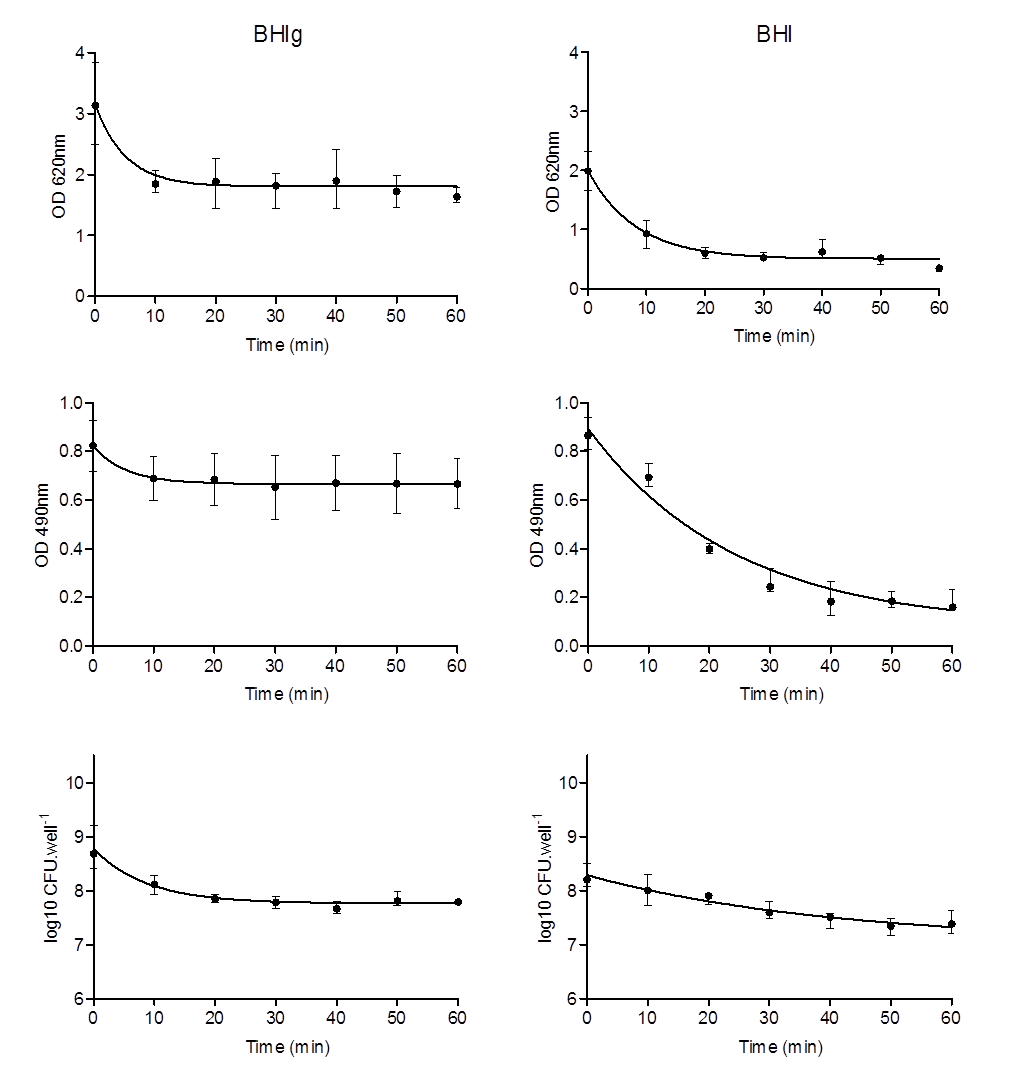


**Supplementary Figure 6. Impact of growth medium on steam wash.** A 24h-old biofilm was formed by SH1000 (*S. aureus)* in BHIg media (a, c, e) or BHI media (b, d, f) and wash using steam method. CV staining and OD_620_ (a, b), PBS turbidity and OD_490_ (c, d) or viable cell count (e, f) was measured every 10 min over 1 h. Data represent the median and interquartile range of three experiments in triplicate.

|  |  | time (min) | | | | |
| --- | --- | --- | --- | --- | --- | --- |
|  |  | **tech 1** | **tech 2** | **tech 3** | **Mean** | **Saving time** |
| 4 wells (1 strain) | **Pipette + CV** | 12 | 10 | 9 | **10.3** |  |
|  | **Steam + PBS** | 11 | 7.5 | 7 | **8.5** | **17.5%** |
| 96 wells | **Pipette + CV** | 25.3 | 33.5 | 21 | **26.6** |  |
|  | **Steam + PBS** | 21.3 | 15.5 | 11 | **15.9** | **40.2%** |

**Supplementary Table 1.** Quantification of the time required to perform crystal violet (CV) staining using pipette washing versus PBS turbidity using steam washing for SH1000 (*S. aureus*) on 4 wells or 96 wells by each technician.
